# The structural and moral integrity of our field

**DOI:** 10.1186/s41687-026-01035-y

**Published:** 2026-04-10

**Authors:** Kevin Weinfurt

**Affiliations:** Department of Population Health Sciences, Duke University School of Medicine, Durham, USA

**Keywords:** Patient-reported outcomes, Validity, Argument-based validity, Research integrity, Health measurement

## Abstract

This commentary consists of edited remarks made by the author upon receipt of the 2025 International Society for Quality of Life Research President’s Award. The field of health measurement/quality-of-life research is viewed as a house in which we have grown up and now live. We sometimes need to rework our foundation to maintain our field’s *structural integrity*—that is, the shared intellectual foundations that support our thinking in a changing world. To complement structural integrity, we need moral integrity—a shared commitment to working in ways that strengthen our science and our humanity. Monitoring and sustaining these two aspects of integrity are the responsibility of everyone.

## Introduction

It is truly an honor to receive ISOQOL’s President’s Award, and I am grateful to the many colleagues and friends in this society who have challenged and encouraged me and who inspire me with their impactful work. This award marks the official beginning of my status as a greying old bastard. And the two main speech options available to greying old bastards are (A) the autobiography and (B) the high-altitude reflection on the field. I chose B.

I’ve spent over 25 years growing up in our field and this society. And I’d like to share two things about my professional home that have become especially important to me in recent years. Now, I’m going to use the metaphor of a home, and I was cautioned by someone not to overdo the metaphor. And it’s true that if you start to push a metaphor too far, you lose people. But I have a hypothesis that this is a nonlinear function, and if I keep pushing this metaphor today, we’ll move from effective to tedious, and then eventually to awesome.

### Structural integrity

As young scholars, none of us had to build a scientific discipline from scratch. We moved into a discipline that was already constructed. And it came pre-furnished with tools and practices that helped us live our day-to-day lives as scientists. The field each of us moved into is supported by a foundation built by those who came before us, using the materials and technology available to them. Parts of the foundation are easy for us to see—things like conceptual frameworks of health and the statistical models we estimate. The foundation also has parts that are hard to see because they’re inside the walls or down in the crawlspace.

With the passage of time, we may start to notice small cracks forming, or that some things don’t feel as comfortable as they used to. We also see new opportunities to renovate or enlarge. In our field, I think of things like the incorporation of more non-Western perspectives on health and quality of life; of types of assessments beyond patient-reported measures; and, as this conference has highlighted, of the potential role of generative AI. When such challenges or opportunities arise, we might need to examine our foundation more closely to determine whether any changes are needed to accommodate them. I’d like to share one example of how this happened in my own work.

About 8 or 9 years ago, I started to feel uneasy. When people asked me about validity, I found myself giving the answers I had been taught—the answers that had worked for me in my career up to that point. But as I listened to my own answers, I had to admit that I no longer truly understood what I was saying. It seemed to me that our approach to validity, a key part of our foundation, was not helping us resolve longstanding uncertainties in our field. The separate types of validity didn’t fit together coherently, and the validity framework provided little guidance—at least to me—on how to make principled decisions about measures.

I started to play with the alternative notion of validity as an argument, like assembling a case in court. I introduced this idea at a meeting, and one of the attendees, Mike Edwards, said (not literally), “Hey, that’s not new. They’ve got something like that over where educational and psychological testing live.” So I decided to check out their house.

I got a hold of the blueprints for their place, the *2014 Standards for Educational and Psychological Testing* [1]. Lo and behold, they did describe validation as a process of constructing arguments for and against proposed interpretations and uses of test scores. Furthermore, I found that there were earlier versions of these blueprints dating back a long way. So I did a little research and found out that our place—health measurement—was built back in the 1970s, following the 1974 blueprints for educational and psychological testing. This was all news to me. (See [2].)

So I read everything I could find on more modern conceptions of validity and started thinking about what changes might be needed to our foundation. I tried to adapt these ideas to health measurement and shared my initial thoughts in a draft manuscript with several colleagues. Karon Cook, Kathryn Flynn, Bryce Reeve, Arthur Stone, and two anonymous reviewers really helped to improve the ideas that were finally published in one of our ISOQOL journals [3] and informed the FDA’s recent guidance on identifying, modifying, and developing fit-for-purpose clinical outcome assessments [4].

Happily, I found a few others in our society who had been looking into formulations of validity developed after the 1970s, including Melanie Hawkins, PhD, in Australia. When I had my first Zoom meeting with Melanie, we discovered that in addition to both being interested in modern validity theory, we had both been gymnasts. So stay tuned for a very niche ISOQOL Special Interest Group. . .

While there may be consternation about changing the foundations, I think people are starting to realize that most of what we were already doing in the name of validity stays the same. We just needed to rearrange, rename, and fortify a few parts. I believe these changes to our foundation will support the wise use of an ever-increasing array of measures.

So, we sometimes need to rework our foundation to maintain our field’s *structural integrity*—that is, the shared intellectual foundations that support our thinking in a changing world. This example highlights one thing we can do to ensure a good foundation for our work: Go visit the neighbors! Psychology, sociology, linguistics, epidemiology, biostatistics, anthropology, philosophy, economics, and clinical medicine—all may have encountered and addressed challenges we face now and have important lessons to teach us.

What else can we do to ensure the structural integrity of our house? Find a little time to familiarize yourself with the blueprints of our discipline by reading some of the great books we have, such as Fayers and Machin [5]; de Vet et al. [6]; and Streiner et al. [7]. I guarantee you will learn something, wherever you are in your career. And you’ll be better positioned to recognize if and when a structural change is needed.

Finally, try to befriend someone with philosophical training. It’s like knowing a structural engineer or a plumber who can put on a helmet and go examine the cramped, dark recesses of your house. One of the most exciting developments in ISOQOL has been the growing presence of philosophers with a deep interest in patient-centered measurement. Get to know them.

### Moral integrity

Structural integrity ensures a solid house. But to be called a home, it needs to be inhabited by people with shared moral commitments. To complement structural integrity, we need moral integrity—a shared commitment to working in ways that strengthen our science and our humanity. What are these shared moral commitments? One obvious commitment is to use science to improve the lives of people suffering from ill health. We all agree that our efforts need to help people suffer less, or else we are not doing our jobs. But I want to talk about some moral commitments we share that may not be so obvious. They might go unnoticed because they’re embedded in the routine aspects of our work, but they are no less profound.

A while back, it was thought that kids weren’t reliable enough to self-report their own symptomatic adverse events in pediatric oncology trials. Then Bryce Reeve, Pam Hinds, and their colleagues showed that this belief was inaccurate, and they developed effective ways for children in those trials to describe their own health experiences [8]. This wasn’t easy, but Bryce and Pam were driven to give children the respect they deserved as they negotiated very serious health challenges. This is a great illustration of our commitment to honoring the experiences and voices of people who are suffering.

As another example, I had the privilege of working with Kathryn Flynn and others to develop the PROMIS measures of sexual function and satisfaction [9]. I knew that we were developing a measure primarily to assess the effects of interventions. But then I began to see that we were doing something else. As we talked to people as part of our qualitative work, I was struck by the sense of shame felt by some people who had sexual challenges brought on by their disease or its treatment. Shame separates a person from others. It’s a second layer of suffering. But when we take care to engage people sensitively about their experience of sexuality—including through our qualitative and quantitative assessments—we are saying that we can talk about these experiences and examine them together. And in doing so, we might help to heal the disconnection caused by shame.

Honoring the experiences and voices of people who are suffering—That’s a shared commitment.

Beyond our commitments to those who are suffering from ill health, we also act out of strong moral commitments to one another as scientists. Over the past year, this set of moral commitments has been especially important to me. Let me give a seemingly small example: Earlier this year, I submitted a commentary to *Quality of Life Research* about interpreting treatment effects. The journal sent it out for review, and some weeks later, I received a message saying it had been accepted with minor revisions based on the feedback from four anonymous reviewers. And though the journal requested only minor revisions, the reviewers’ insights were so substantive and stimulating that I ended up revising half of my commentary [10].

Now, so far, this does not sound like an interesting story. But just look at what happened here: Four scientists, each with their own careers and home lives to manage, volunteered to spend a few hours carefully reading my paper and offering ideas to improve it, all the while knowing that their names would never be attached to any contributions they made. They didn’t get a year-end salary bonus for the ideas they shared with me. They didn’t get hourly consulting money for doing the review. They can’t take credit for the ideas on their CVs. And yet they substantially elevated this scholarly product. It happens so often that we forget that this is quite remarkable.

The reason those reviewers did it—and the reason any of us do—is that we share a commitment to aligning our field’s scientific beliefs with the way the world actually is—toward truth, whether you believe in a small *t* or big *T* truth. We strive to cultivate intellectual humility, recognizing that I might not be right in this moment. And we rely on our community of colleagues to help us get closer to the truth. This is one of the reasons that my old mentor, the late Rom Harré, said that he regarded science as the greatest moral achievement of humankind.

I find that the moral character of our work has become increasingly important to me as I grow older. And it has taken on an extra sense of urgency as I watch parts of our world doubt the value of empirical science and disregard the virtues that have guided scientific work for so long. At the same time, this makes me appreciate the preciousness of this community of scholars and be grateful for the privilege of working every day with colleagues in academia, industry, FDA, NIH, and all who commit their energies to trustworthy science in the service of reducing suffering.

## Conclusion

A good home requires structural and moral integrity. And I feel strongly that monitoring and sustaining these are the responsibility of everyone, from the trainees all the way up to the greying old bastards. Thank you all for making this community a source of inspiration and support for me, and thank you for this award.

## Data Availability

No datasets were generated or analysed during the current study.
